# Use of Lung Ultrasound in the New Definitions of Acute Respiratory Distress Syndrome Increases the Occurrence Rate of Acute Respiratory Distress Syndrome

**DOI:** 10.1097/CCM.0000000000006118

**Published:** 2023-11-13

**Authors:** Coen Plantinga, Peter Klompmaker, Mark E. Haaksma, Amne Mousa, Siebe G. Blok, Micah L.A. Heldeweg, Frederique Paulus, Marcus J. Schultz, Pieter R. Tuinman

**Affiliations:** 1 Department of Intensive Care Medicine, Amsterdam University Medical Centers, location VUmc, Amsterdam, The Netherlands.; 2 Amsterdam Leiden IC Focused Echography (ALIFE, www.alifeofpocus.com), Amsterdam, The Netherlands.; 3 Amsterdam Cardiovascular Sciences Research Institute, Amsterdam UMC, Amsterdam, The Netherlands.; 4 Department of Intensive Care, Amsterdam University Medical Centers, location AMC, Amsterdam, The Netherlands.

**Keywords:** ARDS, lung ultrasound, point of care ultrasound

## Abstract

**OBJECTIVES:**

To assess the effect of incorporating bilateral abnormalities as detected by lung ultrasound (LUS) in the Kigali modification and the New Global definition of acute respiratory distress syndrome (ARDS) on the occurrence rate of ARDS.

**DESIGN:**

Post hoc analysis of a previously published prospective cohort study.

**SETTING:**

An academic mixed medical-surgical ICU.

**PATIENTS:**

The original study included critically ill adults with any opacity on chest radiography in whom subsequent LUS was performed. Patients with ARDS according to the Berlin definition, COVID-19 patients and patients with major thorax trauma were excluded.

**INTERVENTIONS:**

None.

**MEASUREMENTS AND MAIN RESULTS:**

LUS was performed within 24 hours of chest radiography and the presence of unilateral and bilateral abnormalities on LUS and chest radiograph (opacities) was scored. Subsequently, the Kigali modification and the New Global definition of ARDS were applied by two independent researchers on the patients with newly found bilateral opacities. Of 120 patients, 116 were included in this post hoc analysis. Thirty-three patients had bilateral opacities on LUS and unilateral opacities on chest radiograph. Fourteen of these patients had ARDS according to the Kigali modification and 12 had ARDS according to the New Global definition. The detected LUS patterns were significantly different between patients with and without ARDS (*p* = 0.004). An A-profile with a positive PosteroLateral Alveolar and/or Pleural Syndrome was most prevalent in patients without ARDS, whereas heterogeneous and mixed A, B, and C patterns were most prevalent in patients with ARDS.

**CONCLUSION:**

The addition of bilateral abnormalities as detected by LUS to the Kigali modification and the New Global definition increases the occurrence rate of the ARDS. The nomenclature for LUS needs to be better defined as LUS patterns differ between patients with and without ARDS. Incorporating well-defined LUS criteria can increase specificity and sensitivity of new ARDS definitions.

**KEY POINTS**

**Question:** This study investigates whether the addition of bilateral abnormalities detected by lung ultrasound, alongside other criteria, to the Kigali modification and the New Global acute respiratory distress syndrome (ARDS) definition increases the occurrence rate of ARDS.

**Findings:** In a cohort of 116 ICU patients with unilateral opacities on chest radiograph, 14 and 12 new patients met the Kigali modification and New Global ARDS definition, respectively. Bilateral abnormalities found with lung ultrasound were significantly different between patients with and without ARDS.

**Meaning:** The addition of lung ultrasound alongside the diagnostic criteria of the new ARDS definitions increases the occurrence rate of ARDS.

Since the first description of acute respiratory distress syndrome (ARDS) in 1967, researchers have been trying to improve the definition of ARDS as most diagnostic criteria are insensitive and nonspecific (1). The current Berlin definition of ARDS includes bilateral opacities on chest radiography in its definition (1). However, after the introduction of the Berlin definition, lung ultrasound (LUS) is increasingly used to detect pulmonary pathology, and LUS has a higher sensitivity for detecting lung abnormalities than chest radiography (2). In fact, several studies have suggested to use LUS to facilitate the diagnosis of ARDS (3–5). Indeed, in the Kigali modification of the Berlin definition LUS could be used as substitute for chest radiography or CT as it is a cost effective and timely diagnostic tool (3). This has also been adopted in a recently proposed new definition, named the “New Global definition of ARDS” (6). Furthermore, both of these definitions allow patients not on mechanical ventilation to be diagnosed with ARDS, which is especially important in low resource settings. The effect of the addition of LUS to ARDS criteria has been investigated before in ventilated patients (7). However, it is has not been investigated in patients on high flow nasal oxygen therapy or other supplemental oxygen therapy nor has the effect of the New Global definition of ARDS been investigated.

We hypothesize that, in critically ill patients, using LUS to detect bilateral pulmonary abnormalities increases the occurrence rate of ARDS, if applied alongside the other criteria in the Kigali modification and the New Global definition, when compared with using chest radiography. Furthermore, we hypothesize that there is a difference in the detected profiles between ARDS and non-ARDS patients. The primary objective of this study was to assess the effect of using LUS in the Kigali modification and New Global definition on the occurrence rate of ARDS. The secondary objective was to assess whether there is a difference in the LUS profiles of ARDS and non-ARDS patients. To test our hypothesis we will use data of a previously published cohort, which excluded patients with ARDS according to the Berlin definition, to investigate if any new cases of ARDS are present when the Kigali modification and the New Global definition of ARDS are applied.

## METHODS

This is a post hoc analysis of a single-center prospective diagnostic accuracy study using LUS to differentiate pneumonia from atelectasis performed at a mixed medical-surgical ICU at the Amsterdam University Medical Center, location VUmc, Amsterdam, The Netherlands (8).

### Ethics

The original protocol was approved by the Medical Ethics Review Committee of VU Medical Center on October 11, 2016 (protocol number 2016.465 study title: Extended Lung Ultrasound to Differentiate Between Pneumonia and Atelectasis in Critically Ill Patients: A Diagnostic Accuracy Study) and registered in the Dutch Trial Registry (NL 9186). Written informed consent was obtained from all patients or their next of kin. The study procedures were followed in accordance with the committees ethical standards on human experimentation and the Helsinki Declaration of 1975.

### Subjects

The study included critically ill patients younger than 18 with any opacity on chest radiography in whom subsequent LUS was performed within 24 hours (8). Exclusion criteria were the presence of ARDS according to the Berlin definition, COVID-19 patients, and patients with major thorax trauma.

### Measurements

We scored the presence of unilateral and bilateral abnormalities on LUS (B or C pattern, or posterior consolidation) and on chest radiography (opacities) (9). Any patient with bilateral B, C, or mixed patterns was scored as having bilateral abnormalities. Subsequently, the Kigali modification and the “New Global ARDS definition” (**Table 1**) were applied by two independent researchers on the newly found patients with bilateral abnormalities on LUS (3, 6). The two researchers were blinded to each other’s decision and disagreement was resolved by a third researcher.

**TABLE 1.** Acute Respiratory Distress Syndrome Definitions and Their Criteria

	Berlin Criteria	Kigali Modification	New Global Definition of ARDS
Timing	Within 1 wk of a known clinical insult or new or worsening respiratory symptoms	Within 1 wk of a known clinical insult or new or worsening respiratory symptoms	Within 1 wk of a known clinical insult or new or worsening respiratory symptoms
Oxygenation	Pao_2_/Fio_2_ ≤ 300 mm Hg	Spo_2_/Fio_2_ ≤ 315 mm Hg with Spo_2_ < 97% as hypo-oxygenation	Pao_2_/Fio_2_ ≤ 300 mm Hg or Spo_2_/Fio_2_ ≤ 315 mm Hg with Spo_2_ < 97% as hypo-oxygenation when Pao_2_/Fio_2_ is unavailable
PEEP requirement	Minimum 5 cm H_2_O PEEP with invasive or noninvasive in mild ARDS	No PEEP requirement	Minimum 5cm H2O PEEP with invasive or noninvasive in mild ARDS or ≥ 30L high flow nasal oxygen PEEP requirements can be left out in resource poor settings
Chest imaging	Bilateral opacities not fully explained by effusions, lobar/lung collapse, or nodules by chest radiography or CT	Bilateral opacities not fully explained by effusions, lobar/lung collapse, or nodules by chest radiography, CT, or lung ultrasound	Bilateral pulmonary abnormalities not fully explained by effusions, lobar/lung collapse, or nodules by chest radiography, CT, or lung ultrasound
Origin of edema	Respiratory failure not fully explained by cardiac failure or fluid overload	Respiratory failure not fully explained by cardiac failure or fluid overload	Respiratory failure not fully explained by cardiac failure or fluid overload

ARDS = acute respiratory distress syndrome, PEEP = positive end expiratory pressure.

### Statistical Analysis

Continuous variables are expressed as mean (± sd) or as a median (interquartile range) depending on distribution. The McNemar test was used to assess the difference in occurrence rate of bilateral abnormalities between LUS and chest radiography. New cases of ARDS were described as a percentage of the patient population. For the secondary outcome, a chi-square test was used to assess the occurrence rate of the different LUS profiles between non-ARDS and ARDS patients.

## RESULTS

Of 120 patients, 116 were included in this post hoc analysis. Four patients were excluded due to missing data. The mean age was 63 (± 16) years, 68% was male, mean Sequential Organ Failure Assessment score was 9 (± 3), and the most important reason for admission was cardiovascular disease.

LUS detected bilateral abnormalities more often than chest radiography (in 86.2% versus 60.3% of the patients; *p* < 0.001). Thirty-three patients (28.4%) with only unilateral opacities on chest radiography had bilateral abnormalities on LUS. Three patients (2.6%) with bilateral opacities on chest radiography had only unilateral abnormalities on LUS (**Supplemental Fig. 1**, http://links.lww.com/CCM/H458).

Fifteen of the 33 patients with bilateral abnormalities on LUS and unilateral opacities on chest radiography had a Pao_2_/Fio_2_ ratio of greater than or equal to 300 mm Hg or a saturation of greater than 97%, and four patients had their respiratory insufficiency explained by lung collapse. Using the Kigali modification and New Global ARDS definition in patients with new bilateral abnormalities found by LUS, 14 of 116 patients (12%) and 12 of 116 patients (10%) were diagnosed with ARDS, respectively (**Table 2**). One patient who met the New Global definition did not meet the Kigali modification due to a too high Spo_2_. Of 19 patients without ARDS with bilateral abnormalities on LUS 15 patients (83%) had an A-profile with a bilaterally positive PosteroLateral Alveolar and/or Pleural Syndrome (PLAPS) and 3 patients (17%) had a combination of A, B, and C patterns. Of 15 patients with ARDS and bilateral abnormalities on LUS 4 patients (27%) had an A profile with positive PLAPS, 2 patients (13%) had a combination of B and C patterns, and 9 patients (60%) had a combination of A, B, and C patterns. The detected LUS patterns were significantly different between non-ARDS and ARDS patients (*p* = 0.004). Of 116 patients, 20 (17%) died within 28 days of inclusion. Mortality in patients without ARDS was 18% versus 14% (Kigali) and 17% (New Global).

**TABLE 2.** Lung Ultrasound Findings in Patients With Bilateral Abnormalities on Lung Ultrasound but Unilateral Opacities on Chest Radiograph and With Acute Respiratory Distress Syndrome (ARDS) Diagnosis According to the Kigali Modification or the New Global Definition of ARDS

Variable	Total (*N* = 33)	Kigali Modification (*N* = 14)	New Global Criteria (*N* = 12)
Imaging			
Chest radiography (unilateral)^a^			
Infiltrate/consolidation	17 (51.5%)	10(71.4%)	8(66.7%)
Pleural effusion	11 (33.3%)	4(28.6%)	1(8.3%)
Atelectasis	13 (39.4%)	3(21.4%)	4(33.3%)
Lung ultrasound (bilateral)^b^			
A-profile with positive PosteroLateral Alveolar and/or Pleural Syndrome^c^	19 (57.6%)	3 (21.4%)	4(33.3%)
Combination of B and C patterns^c,d^	2 (6.1%)	2 (14.3%)	1 (8.3%)
Combination of A, B, and C patterns^c,d^	12 (36.4%)	9 (64.3%)	7 (58.3%)
Respiratory			
Invasively ventilated	16 (48.4%)	9 (64.3%)	9 (75.0%)
High flow nasal oxygen ≥ 30 L/min	5 (15.2%)	2 (14.3%)	3 (25%)
Low flow oxygen < 30 L/min	12 (36.4%)	3 (21.4%)	0 (0%)
Positive end expiratory pressure ≥ 5 cm H_2_O	15 (54.5%)	NA	9 (75.0%)
Pao_2_/Fio_2_ ratio < 300 mm Hg	17 (51.5%)	NA	12 (100%)
Spo_2_/Fio_2_ ratio < 315 mm Hg	22 (66.7%)	14 (100%)	12 (100%)
28 d mortality	3 (9%)	2 (14%)	2 (17%)

Values are presented as mean (± sd) or as number (%).

a Multiple simultaneous opacities possible.

b There were no patients with only bilateral B patterns indicating cardiogenic edema.

c According to BLUE protocol (9).

d B pattern indicates ≥ 3 B lines in anterior or lateral views; C pattern indicates consolidation in anterior or lateral views.

## DISCUSSION

The findings of this post hoc analysis provides evidence that the use of LUS in the Kigali modification and New Global criteria increases the occurrence rate of ARDS when compared with using chest radiography to diagnose bilateral opacities. Furthermore, we found that there is a significant difference in the LUS patterns between patients with and without ARDS.

The higher occurrence rate of bilateral abnormalities detected by LUS compared with chest radiography we found confirms previous literature (2). A recent study in patients with COVID-19 found similar results when comparing LUS and chest radiography (10). Another study has investigated the effect of the Kigali criteria on the occurrence rate of ARDS in ventilated patients only (7). They found 17 new patients with ARDS in a sample of 152 patients. This is comparable to the 14 new patients found in our study. Our results confirm that using LUS for the diagnosis of ARDS will increase the detection and occurrence rate of ARDS and thereby occurrence rate. The occurrence rate of ARDS is further increased by the inclusion of nonventilated patients, five (Kigali) and three (New Global) in the ARDS diagnosis.

The proposed change of adding bilateral abnormalities detected by LUS in the ARDS definition can have several effects. Due to LUS’ higher sensitivity, cases which might not have been recognized as ARDS previously are classified as ARDS now. This might lead to earlier recognition and more adequate treatment.

Furthermore, the Kigali modification uses bilateral opacities in their definition. This term is not generally used in ultrasound imaging. By lumping together all ultrasound abnormalities as opacities specificity in the ARDS diagnosis might be lost. This might increase difficulties in research, finding new treatment strategies and identifying patients most in need for ICU treatment. Although the New Global definition of ARDS does not use the term opacities careful consideration should nonetheless be given to which ultrasound findings are considered as opacities in the ARDS definition. For example, pneumonia and atelectasis can look very similar on ultrasound unless a more extensive protocol is used (8). Nonetheless, LUS has the advantage of being able to differentiate between cardiac and noncardiac pulmonary edema, which can be of benefit in diagnosing ARDS (11). Defining clear LUS criteria for ARDS diagnosis is therefore needed. Especially since there appears to be a difference in the types of profiles detected in patients with and without ARDS.

Our current study has several limitations. First, it has a post hoc design. Second, because the original study excluded patients with ARDS according to the Berlin definition we cannot truly determine the precise effect of using LUS on the occurrence rate of ARDS according to the new criteria. Nonetheless, finding ARDS cases where there were previously no cases makes it likely that the occurrence rate of ARDS would increase. Our results therefore serve as a foundation for future prospective trials.

In conclusion, our results show that incorporating LUS in the ARDS definition increases the occurrence rate of ARDS and that there is a difference in LUS patterns between patients with and without ARDS. Further specification of LUS nomenclature and protocols used is needed to improve the LUS criteria for ARDS and thereby ARDS definition.

## Supplementary Material
