# Supplementary figures and images for: Use of Lung Ultrasound in the New Definitions of Acute Respiratory Distress Syndrome Increases the Occurrence Rate of Acute Respiratory Distress Syndrome
Source: Crit Care Med. 2023 Nov 13;52(2):e100–4. doi: 10.1097/CCM.0000000000006118 (PMC10793806; doi:10.1097/CCM.0000000000006118)

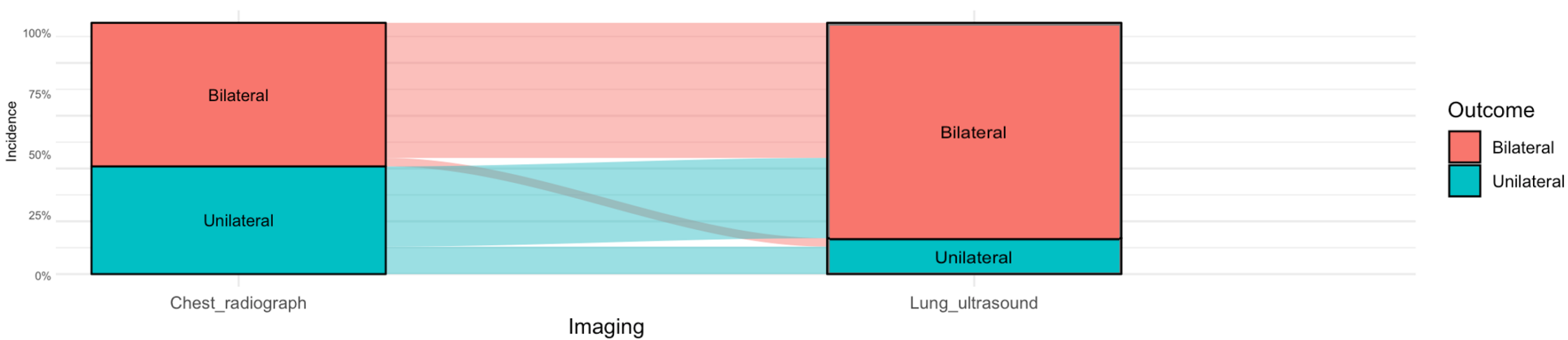

Supplement: Supplementary file 1 [file ccm-52-e100-s001.pdf]
